# Association Between Sleep Duration and Parkinson’s Disease Varied Across Related Orphan Receptor A rs2028122 Genotypes

**DOI:** 10.3389/fnins.2022.902895

**Published:** 2022-06-13

**Authors:** Yuan Shao, Xi-jian Dai, Jian Wang, Yongjun Wang

**Affiliations:** Shenzhen Mental Health Centre, Shenzhen Kangning Hospital, Shenzhen, China

**Keywords:** Parkinson’s disease, sleep duration, RORA, rs2028122, UK Biobank

## Abstract

**Background:**

The purpose of the study was to examine the association of long and short sleep duration with risk of Parkinson’s disease (PD) across RORA rs2028122 genotypes.

**Methods:**

In the present prospective study with a large sized UK Biobank cohort, we performed multivariate logistic regression analyses, generalized additive model, interaction terms, stratification analysis, and mediation analysis to evaluate the association of long and short sleep duration with risk of PD across RORA rs2028122 genotypes.

**Results:**

The GG genotype [1.16 (1.01, 1.33)], a short sleep duration [1.23 (1.10, 1.37)], and a long sleep duration [1.19 (1.03, 1.37)] were identified as the independent risk factors for PD. Sleep duration exhibited a curvilinear U-shaped correlation with the risk of PD; first, the risk of PD gradually decreased as the length of sleep increase, but then, the risk began to increase as the length of sleep increase. Among habitual long sleepers, AG carriers had a higher risk of PD compared with AA carriers [1.67 (1.09, 2.55)]. Among AG carriers, both habitual short [1.28 (1.09, 1.50)] and long [1.38 (1.13, 1.69)] sleepers increased the risk of PD compared with habitual normal sleepers. Among GG carriers, habitual short sleepers have a higher risk of PD [1.26 (1.06, 1.50)] compared with habitual normal sleepers. A mediation model suggested that the rs2028122 genotype partially mediated the causal pathway of sleep duration leading to the development of PD on a positive effect.

**Conclusion:**

Our study demonstrated that the association between sleep duration and PD risk varied across different RORA rs2028122 genotypes. Our findings could help individuals to identify their potential risk profile and take timely actions to prevent the PD.

## Introduction

Previous studies demonstrated that about 64% of patients with Parkinson’s disease (PD) suffered from sleep problems (e.g., short and long sleep duration) (Barone et al., 2009; Mantovani et al., 2018; Doręgowska and Rudzińska-Bar, 2019). Although there has been a notable increase in the number of studies investigating the association between sleep duration and PD, it has not gleaned a consistent conclusion. Previous studies have shown that participants who were habitual short sleepers were associated with a higher risk of developing PD; however, this association was not observed for participants who were habitual long sleepers (Kay et al., 2018; Lysen et al., 2019). In contrast, Chen reported that habitual long sleep duration was associated with a higher risk of PD, but habitual short sleep duration did not increase this risk (Chen et al., 2006). However, these studies were based on a relatively small sample size, and analyses of these studies were not controlled for sufficient confounders (e.g., sleep-related covariates). Therefore, there is a clear need to set up a specific study, with a larger sample size and more confounders, to investigate whether and how long and/or short sleep duration affect the risk of PD.

The retinoic acid receptor-related orphan receptor A (RORA) gene is known to play a key role in circadian rhythm and sleep disorders (Möller-Levet et al., 2013) and was considered as a promising candidate gene for regulating sleep duration (Carvalho et al., 2020; Hou et al., 2020) and sleep latency (Kripke et al., 2015). *De novo* dominant toxic RORA gene variants have found to be associated with ataxia and cerebellar atrophy (Guissart et al., 2018). Knockout of the RORA gene in mice could lead to certain symptoms that are commonly seen in patients with PD, including the tremor and body imbalance (Dussault et al., 1998). These studies present direct evidence for a link of the RORA gene (e.g., rs2028122) with sleep duration and PD.

Therefore, we hypothesized that the RORA gene may play a role in the regulation of sleep duration and PD, and that the association between short and long sleep duration and risk of PD may vary across different RORA genotypes (e.g., rs2028122 genotypes). To test the hypothesis, in the present large observational study with one of the largest cohort worldwide: the UK Biobank, we specifically investigated the association between RORA rs2028122 genotypes, sleep duration, and PD. First, we used multivariate logistic regression analyses and a generalized additive model to evaluate the association of sleep duration and RORA rs2028122 genotypes with the risk of PD. Second, we used interaction terms and stratification analyses to investigate the potential interactions between rs2028122 genotypes and sleep duration on the risk of PD. Finally, mediation analysis was performed to evaluate whether the rs2028122 genotypes mediated in the causal path of sleep duration leading to the development of PD.

## Materials and Methods

### Study Population

We analyzed the data of 502,505 participants from 22 centers between March 2006 and December 2010 in the UK Biobank (Table 1), whom were classified into a PD group (74.85 ± 5.51 years; 61.5% men) and a non-PD group (68.42 ± 8.11 years; 45.5% men). The definition of PD included Parkinson’s disease, secondary Parkinsonism, and Parkinsonism, according to the International Classification of Diseases, version 10 (ICD-10) terms from the UK Biobank data field 41,270 (ICD-10 codes G20-G22). A total of 3,163 (0.63%) PD events were observed during follow-up appointments up to 30 December 2020, which was higher than the prevalence rate of 0.1–0.3% that were reported among the general population (von Campenhausen et al., 2005; Pringsheim et al., 2014; Elbaz et al., 2016). Ethical approval was obtained from the Northwest Multi-Centre Research Ethics Committee (REC reference: 16/NW/0274).

**TABLE 1:** Baseline characteristics of the UK Biobank population, separated by Parkinson’s disease.

Characteristics	PD (*n* = 3,163)	Non-PD (*n* = 499,342)	*p*-*value*
Age categories (years), N (%)			<0.001
≤60	79 (3.7)	104,598 (20.9)	
61–70	479 (15.1)	161,044 (32.3)	
≥71	2,605 (82.4)	233,699 (46.8)	
Sex (male), N (%)	1,944 (61.5)	227,178 (45.5)	<0.001
Education (degree), N (%)	856 (27.9)	160,307(32.8)	<0.001
Ethnicity (white), N (%)	3,030 (96.5)	469,665 (94.5)	<0.001
Obesity (BMI ≥ 30 kg/m^2^), N (%)	813 (26.1)	121,434 (24.5)	0.037
Overall health rating, N (%)			<0.001
Excellent or good	1,660 (53.0)	369,262 (74.4)	
Fair or poor	1,470 (47.0)	126,755 (25.6)	
Employment (in paid), N (%)	806 (25.7)	286,343 (57.7)	<0.001
Smoking (Previous or current), N (%)			0.53
Never	1,698(54.2)	271,824 (54.8)	
Previous or current	1,435 (45.8)	224,599 (45.2)	
Frequency of alcohol consumption, N (%)			<0.001
Never	835 (26.5)	97,814 (19.6)	
Low	1,035 (32.9)	184,112 (37.0)	
High	1,276 (40.6)	215,931 (43.4)	
IPAQ			<0.001
Low	561 (22.8)	75,658 (18.9)	
Moderate	1,050 (42.6)	162,969 (40.8)	
High	852 (34.6)	161,290 (40.3)	
Summed MET minutes per week for all activity (minutes/week), N (%)			<0.01
Low (0–599)	557 (22.6)	75,081 (18.8)	
Moderate (600–1,199)	478 (19.4)	70,566 (17.7)	
High (≥1,200)	1,428 (58.0)	254,142 (63.6)	
Self-report: Sleep duration categories (hours), N (%)			<0.001
Short sleep duration (≤6)	861 (27.6)	122,391 (24.7)	
Normal sleep duration (7–8)	1,839 (58.9)	334,851 (67.6)	
Long sleep duration (≥ 9)	420 (13.5)	37,927 (7.7)	
Actigraphy: Sleep duration categories (hours), N (%)			0.001
Short sleep duration (≤6)	160 (41.8)	34,951 (35.6)	
Normal sleep duration (7–8)	136 (35.5)	44,135 (44.9)	
Long sleep duration (≥9)	87 (22.7)	19,218 (19.5)	
Getting up easily in morning, N (%)			<0.001
Not very easy/Not at all easy	640 (20.6)	89,058 (18.0)	
Fairly easy	1,389 (44.7)	245,257 (49.7)	
Very easy	1,076 (34.7)	159,411 (32.3)	
Morning/evening person (chronotype), N (%)			0.002
Morning person	823 (30.1)	119,541 (27.1)	
Intermediate type	939 (34.3)	156,438 (35.4)	
Evening person	973 (35.6)	165,470 (37.5)	
Nap during day, N (%)			<0.001
Never/rarely	1,236 (39.2)	279,833 (56.3)	
Sometimes	1,483 (47.1)	191,172 (38.4)	
Usually	431 (13.7)	26,455 (5.3)	
Daytime dozing/sleeping (narcolepsy), N (%)			<0.001
Never/rarely	1,907 (61.0)	376,799 (76.0)	
Sometimes	983 (31.5)	104,986 (21.2)	
Often/All of the time	235 (7.5)	13,860 (2.8)	
Sleeplessness or insomnia, N (%)			<0.001
Never/rarely	704 (22.4)	120,071 (24.1)	
Sometimes	1,403 (44.6)	237,434 (47.7)	
Usually	1,040 (33.0)	140,349 (28.2)	
Snoring, N (%)			0.035
Yes	1,151 (39.1)	172,209 (37.2)	
No	1,791 (60.9)	290,301 (62.8)	
RORA rs2028122 genotype, N (%)			0.007
AA	438 (14.4)	79,311 (16.4)	
AG	1,434 (47.3)	228,396 (47.2)	
GG	1,161 (38.3)	176,213 (36.4)	

*Missing data were not shown in this table. Missing data were not analyzed. PD, Parkinson’s disease; N, number; BMI, body mass index; IPAQ, International Physical Activity Questionnaire; MET, metabolic equivalent task; RORA, retinoic acid receptor-related orphan receptor A.*

### Exposure Measures

Participants were genotyped on the Affymetrix UK Biobank Lung Exome Evaluation (UK BiLEVE) Axiom array or the Applied Biosystems UK Biobank Axiom Array. Quality control and imputation, using the Haplotype Reference Consortium, UK10K and 1000 Genomes phase 3, and reference panels were conducted centrally at the UK Biobank, thus resulting in a total of 96 million SNPs (Bycroft et al., 2018). The RORA rs2028122 SNP (located on chromosome 15) was among the directly genotyped SNPs from the UK Biobank, including AA, AG, and GG genotypes. The minor allele frequency of the rs2028122 G risk allele was 59.1% in European.

To investigate the sleep duration (ID 1160), participants were asked to answer the question “About how many hours sleep do you get in every 24 h? (Please include naps)” with a response by an exact value. “Do not know” or “Prefer not to answer” responses were considered as missing values. The self-reported sleep duration was classified into three categories for analysis: a short sleep duration (≤6 h), a normal sleep duration (7–8 h), and a long sleep duration (≥9 h). Participants who usually reported short, normal, and long sleep duration were considered as habitual short, normal, and long sleepers, respectively.

### Confounders

Confounding factors included the following: demographic variables b[age (ID 21022), sex (ID 31), educational level (ID 6138), body mass index (BMI) (ID 21001), overall health rating (ID 21780), employment (ID 20277)], lifestyles [smoking (ID 20116), frequency of alcohol consumption (ID 20117), international physical activity questionnaire (IPAQ) (ID 22032), summated metabolic equivalent task (MET) minutes per week for all activity (ID 22040)], and sleep variables [getting up easily in the morning (ID 1170), chronotype (ID 1180), nap during the day (ID 1190), narcolepsy (ID 1220), sleeplessness or insomnia (ID 1200), snoring (ID 1210)]. Educational level (ID 6138) was classified into two categories depending on whether the patient held a college or university degree. Obesity was defined as a body mass index (BMI) ≥ 30 kg/m^2^. Alcohol consumption was divided into a low frequency (<3 times a week), a high frequency (≥3 times a week), and never (never and special occasions only). The overall health rating (ID 21780) was classified into excellent/good and fair/poor health rating.

### Activity Monitor-Derived Measures of Sleep

Actigraphy devices (Axivity AX3) were worn 2.8–9.7 years after the study baseline from the UK Biobank for up to 7 days. Samples were excluded if they satisfied at least one of the following conditions: a non-zero or missing value in data field 90002 (data problem indicator), good wear time flag (field 90015) set to 0, good calibration flag (field 90016) set to 0, calibrated on own data flag (field 90017) set to 0, or overall wear duration (field 90051) less than 5 days. Finally, 98765 samples remained.

Sleep episodes within the sleep period time window were defined as periods of at least 5 min with no changes larger than 5° associated with the *z*-axis of the accelerometer. The summed duration of all sleep episodes was used as an indicator of sleep duration.

### Statistical Analysis

Categorical variables are presented as a number (percentage). We used unpaired *t*-tests, Mann–Whitney *U*-tests, and χ^2^-tests to compare the differences between groups where appropriate.

Logistic regression analysis was used to evaluate the associations of exposure variables (the rs2028122 genotype and sleep duration) with the risk of PD. Univariate and multivariate models were applied to evaluate the odds ratios (ORs) and 95% confidence intervals (95% CIs). A total of four schemes were analyzed by logistic regression analysis: (a) model 1, adjusted for age and sex; (b) model 2, further adjusted for education level, ethnicity, BMI, overall health rating, frequency of alcohol consumption, and employment status; (c) model 3, further adjusted for IPAQ and summated MET minutes per week for all activities; and (d) model 4, further adjusted for sleep variables. To compare the goodness of fit between statistical models, we calculated the Nagelkerke’s R-squared value for each logistic regression model.

Next, the sleep duration was treated as a continuous variable. We used penalized cubic splines in a generalized additive model (GAM) to evaluate the non-linear association between sleep duration and the risk of PD. Non-linearity was evaluated using the likelihood ratio test. If a *p*-value for non-linearity was < 0.05, it indicated the existence of evidence against the linearity assumption.

Furthermore, interaction terms were employed for the overall sample to explore potential interactions between the rs2028122 genotype and the sleep duration (“rs2028122 genotype * sleep duration”) on the risk of PD after controlling for demographic variables, lifestyles, and sleep variables. Mediation analysis was used to evaluate whether the rs2028122 genotype mediated the causal path of sleep duration leading to the development of PD.

All analyses were conducted with SPSS version 24.0 and R statistical software version 4.0. A two-tailed *p*-value < 0.05 was considered significant.

## Results

### Sample Characteristics

The demographic characteristics of the study population are presented in Table 1. Compared with non-PD group, PD group had a significant higher proportion of G risk allele (χ^2^ = 9.87, *p* = 0.007), and that of short/long sleep duration (χ^2^ = 180.60, *p*< 0.001). Compared with non-PD group, PD group was characterized by a lower educational level, a higher proportion of obesity, a poorer overall health rating, a poorer employment status, a lesser physical activity, and more sleep problems. The distribution of sleep duration across different rs2028122 genotypes is shown in Table 2 (χ^2^ = 108.129, *p* < 0.001).

**TABLE 2:** Crosstab between sleep duration categories and RORA rs2028122 genotype.

	rs2018122 genotype		

**Sleep duration categories**	**AA**	**AG**	**GG**
Short sleep duration (≤6), n (%)	20,646 (17.3)	56,070 (47.0)	42,684 (35.7)
Normal sleep duration (7–8), n (%)	52,408 (16.0)	154,562 (47.3)	119,826 (36.7)
Long sleep duration (≥9), n (%)	5,976 (16.1)	17,570 (47.4)	13,526 (36.5)

### Non-linear Associations

**FIGURE 1:**
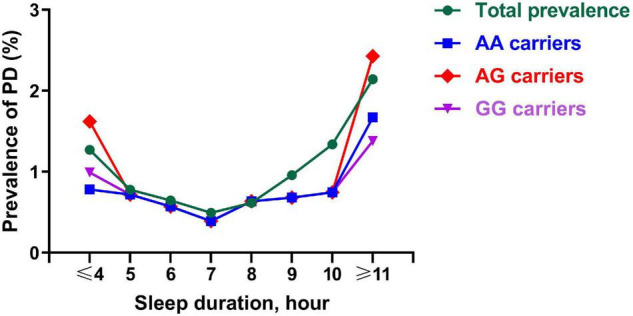
Prevalence of PD across a different sleep length spectrum. Each node represents the prevalence of PD of the corresponding sleep duration. This figure shows a curvilinear U-shaped correlation of sleep duration with prevalence of PD for AA carriers (blue line), AG carriers (red line), GG carriers (purple line), and total subjects (green line). PD, Parkinson’s disease.

The prevalence of PD exhibited a curvilinear U-shaped correlation with the length of sleep (Figure 1), which suggests that the prevalence of PD gradually decreased as the increase of sleep length, and then, it began to increase as the increase of sleep length at the inflexion about 7 h. Carriers of AG genotype had the highest incidence of PD among the rs2028122 genotypes at both ends of the sleep length spectrum.

**FIGURE 2:**
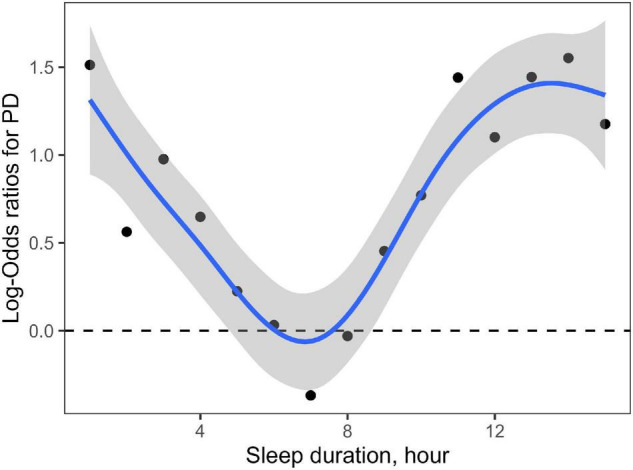
Non-linear association of sleep duration with risk of PD. Each node represents the logarithm of OR value of the corresponding sleep duration increasing the risk of PD. The shaded area represents the 95% confidence interval of the logarithm of OR value. This figure shows a curvilinear U-shaped correlation of sleep duration with the risk of PD.

A generalized additive model showed a non-linear association between the sleep duration and the risk of PD (Figure 2). Sleep duration also exhibited a curvilinear U-shaped correlation with the risk of PD, which was as the same as sleep duration with the prevalence of PD.

### Interaction Effects Between rs2028122 Genotypes and Sleep Duration on the Risk of Parkinson’s Disease

Table 3 shows that there was a significant interaction effect between the RORA rs2028122 AG genotype and long sleep duration (AG genotype * long sleep duration) regarding the risk of PD without adjusting for any confounders [1.44 (1.01–2.05), *p* = 0.042]. This interaction effect remained significant after adjusting for age, sex, education level, employment status, ethnicity, BMI, overall health rating, lifestyles, and sleep-related factors [1.73 (1.10–2.72), *p* = 0.019]. However, there is no interaction effect between AG genotype and short sleep duration [1.23 (0.90–1.68), *p* = 0.19], between GG genotype and long sleep duration [1.48 (0.93–2.36), *p* = 0.10], and between GG genotype and short sleep duration [1.24 (0.91–1.71), *p* = 0.18].

**TABLE 3:** Interaction effect between rs2028122 genotype and sleep duration on PD risk.

(a)	Sleep duration		

**rs2028122 genotype**	**Normal sleep duration**	**Short sleep duration**	**Long sleep duration**
AA	1	1	1
AG	1	1.06 (0.83–1.35)	1.44 (1.01–2.05)
GG	1	1.06 (0.83–1.37)	1.24 (0.86–1.78)

**(b)**	**Sleep duration**		

**rs2028122 genotype**	**Normal sleep duration**	**Short sleep duration**	**Long sleep duration**
AA	1	1	1
AG	1	1.23 (0.90–1.68)	1.73 (1.10–2.72)
GG	1	1.24 (0.91–1.71)	1.48 (0.93–2.36)

*Interaction effect was performed for all samples in this table. (a) Odds ratios and 95% CIs for the interaction effect between rs2028122 genotype and sleep duration on PD risk without adjusting for any confounders. (b) Odds ratios and 95% CIs for the association between rs2028122 genotype and sleep duration on the risk of PD, after adjusting for age, sex, education, ethnicity, BMI, overall health rating, employment, alcohol intake frequency, IPAQ, summed MET minutes per week for all activity, getting up easily in morning, chronotype, narcolepsy, insomnia, snoring. CI, confidence interval; PD, Parkinson’s disease; BMI, body mass index; IPAQ, International Physical Activity Questionnaire; MET, metabolic equivalent task.*

### Associations Between Related Orphan Receptor A rs2028122 Genotypes, Sleep Duration, and Parkinson’s Disease

The associations between the RORA genotype, sleep duration, and the risk of PD are presented in Table 4. Multivariate logistic regression model showed that compared with participants who carried the rs2028122 AA genotype, those GG carries had a higher risk of PD [OR (95% CI), 1.16 (1.01, 1.33), *p* = 0.038, Model 4]. The AG genotype was not associated with a higher risk of PD [1.07 (0.93, 1.22), *p* = 0.35]. Compared with participants who usually reported a normal sleep duration, those habitual long sleepers [1.19 (1.03, 1.37), *p* = 0.019] and habitual short sleepers [1.23 (1.10, 1.37), *p*< 0.001] were associated with a higher risk of PD.

**TABLE 4:** Odds ratios and 95% CIs of rs2028122 genotype and sleep duration for Parkinson’s disease.

	Model 1			Model 2			Model 3			Model 4		
Exposure	OR (95% CI)	P*	*R* ^2^	OR (95% CI)	p*	*R* ^2^	OR (95% CI)	p*	*R* ^2^	OR (95% CI)	p*	*R* ^2^
**rs2028122 genotype**												
AA	1		0.06	1		0.08	1		0.09	1		0.10
AG	1.10 (0.99–1.23)	0.07		1.11 (0.99–1.24)	0.07		1.06 (0.94–1.20)	0.34		1.07 (0.93–1.22)	0.35	
GG	1.16 (1.04–1.29)	0.009		1.17 (1.05–1.31)	0.006		1.14 (1.01–1.29)	0.042		1.16 (1.01–1.33)	0.038	
**Sleep duration**												
Normal sleep duration	1		0.06	1		0.09	1		0.09	1		0.10
Short sleep duration	1.32 (1.22–1.43)	<0.001		1.18 (1.09–1.29)	<0.001		1.19 (1.09–1.31)	<0.001		1.23 (1.10–1.37)	<0.001	
Long sleep duration	1.69 (1.52–1.88)	<0.001		1.31 (1.17–1.46)	<0.001		1.27 (1.12–1.45)	<0.001		1.19 (1.03–1.37)	0.019	

*Multivariate logistic regression was performed for all sample in this table.*

*Model 1: adjusted for age and sex.*

*Model 2: adjusted for confounders in Model 1 + education + ethnicity + BMI + overall health rating + alcohol intake frequency + employment.*

*Model 3: adjusted for confounders in Model 2 + IPAQ + Summed MET minutes per week for all activity.*

*Model 4: adjusted for confounders in Model 3 + self-reported sleep duration (or rs2028122 genotype when investigating the association between self-reported sleep duration and Parkinson’s disease) + getting up easily in morning + chronotype + narcolepsy + insomnia + snoring.*

*Compared to the reference group (derived from logistic regression analysis). To compare the goodness of fit between statistical models, we computed the Nagelkerke’s R-squared value for each logistic regression model. A higher R-squared value reflects better fitness of the logistic regression Model. CI, confidence interval; ORs, odds ratios.

### Stratification Analysis

We investigated the association between the RORA rs2028122 genotypes and the risk of PD, separated by sleep duration (Table 5a) and RORA rs2028122 genotype (Table 5b), respectively. When stratifying our analysis by sleep duration, we found that among habitual long sleepers, AG genotype increased the risk of PD compared with AA genotype [1.67 (1.09, 2.55), *p* = 0.019]. In contrast, among normal [0.96 (0.81, 1.13), *p* = 0.60] and short [1.13 (0.87, 1.47), *p* = 0.35] sleepers, AG genotype was not associated with the PD risk. Among habitual normal sleepers, the GG genotype was not associated with the risk of PD [1.06 (0.89, 1.26), *p* = 0.51].

**TABLE 5:** Odds ratios and 95% CIs for the association between RORA rs2028122 genotype and PD, separated by sleep duration and RORA rs2028122 genotype, respectively.

	Sleep duration					
rs2028122 genotype	Normal sleep duration OR (95% CI)	*p*	Short sleep duration OR (95% CI)	*p*	Long sleep duration OR (95% CI)	*p*
**(a)**						
AA	1		1		1	
AG	0.96 (0.81–1.13)	0.60	1.13 (0.87–1.47)	0.35	1.67 (1.09–2.55)	0.019
GG	1.06 (0.89–1.26)	0.51	1.27 (0.97–1.66)	0.082	1.50 (0.97–2.33)	0.069

	**rs2028122 genotype**					
**Sleep duration categories**	**AA OR (95% CI)**	***p***	**AG OR (95% CI)**	***p***	**GG OR (95% CI)**	***p***

**(b)**						
Normal sleep duration	1		1		1	
Short sleep duration	0.99 (0.75–1.33)	0.97	1.28 (1.09–1.50)	0.003	1.26 (1.06–1.50)	0.009
Long sleep duration	0.79 (0.52–1.20)	0.27	1.38 (1.13–1.69)	0.001	1.11 (0.89–1.40)	0.36

*Stratification analysis was performed for all sample in this table. (a) Odds ratios and 95% CIs for the association between RORA rs2028122 genotype and PD, separated by sleep duration. (b) Odds ratios and 95% CIs for the association between sleep duration and Parkinson’s disease, separated by the RORA rs2028122 genotype. Logistic regression analysis was adjusted for age, sex, education, ethnicity, BMI, overall health rating, alcohol intake frequency, employment, IPAQ, summed MET minutes per week for all activity, self-reported sleep duration, getting up easily in morning, chronotype, narcolepsy, insomnia, snoring, and rs2028122 genotype. CI, confidence interval; ORs, odds ratios.*

When stratifying our analysis by RORA rs2028122 genotype, we found that among AG carriers, compared with habitual normal sleepers, habitual short sleepers [1.28 (1.09, 1.50), *p* = 0.003] and habitual long sleepers [1.38 (1.13, 1.69), *p* = 0.001] were associated with a higher risk of PD, respectively. Among GG carriers, only habitual short sleepers were associated with a higher risk of PD [1.26 (1.06, 1.50), *p* = 0.009].

### Sensitivity Analyses on Association Between Sleep Duration and Parkinson’s Disease

We also analyzed the association of sleep duration with the risk of PD among those participants who were worn the actigraphy devices by multivariate logistic regression, interaction terms, and stratification analysis after adjusting for all confounders. We found a consistent finding of the association of sleep duration with the risk of PD. Compared with habitual normal sleepers, long [1.46 (1.09, 1.97), *p* = 0.012] and short [1.54 (1.19, 2.00), *p* = 0.001] sleepers were also associated with a higher risk of PD.

When stratifying our analysis by RORA rs2028122 genotype, we found that among the AG carriers, compared with habitual normal sleepers, those habitual short [1.87 (1.27, 2.74), *p* = 0.002] and long [1.79 (1.16, 2.77), *p* = 0.009] sleepers were associated with a higher risk of PD, respectively.

### Mediating Causality Analysis

**FIGURE 3:**
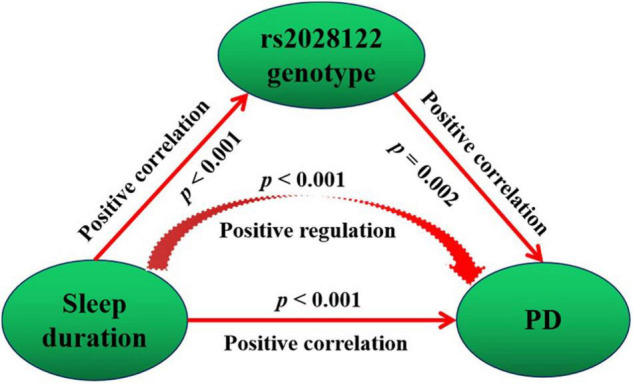
Mediating causality of the RORA rs2028122 in the causal path of sleep duration leading to PD. Mediation model suggests that the rs2028122 genotype partially mediates the causal path of sleep duration leading to development of PD on positive effect.

The mediation model suggests that the rs2028122 genotype partially mediates the causal pathway of sleep duration leading to the development of PD on a positive effect (Figure 3). In the mediation model, the average causal mediation effect (*p* = 0.002), average direct effect (*p* = 0.002), and total effect (*p* < 0.001) were all statistically significant.

## Discussion

This prospective study of a large-sized UK Biobank cohort was the first epidemiological study to investigate whether (and how) sleep duration and RORA rs2028122 genotype could affect the risk of PD. Our study identified five main novel findings. First, the GG genotype, a short sleep duration, and a long sleep duration were identified as the independent risk factors for PD. Second, the RORA rs2028122 genotype mutation did not increase the risk of PD among normal sleepers; furthermore, neither a long nor a short sleep duration increased the risk of PD among AA carriers. Third, the RORA G risk allele increased the risk of PD. Short sleepers had a 26% higher risk of PD among GG carriers. Short sleepers and long sleepers had 28 and 38% higher risk of PD among AG carriers, respectively. Fourth, we identified a significant interaction effect between the AG genotype and a long sleep duration (there was a 73% higher risk of PD). Furthermore, the AG genotype increased 67% risk of PD among those habitual long sleepers. Finally, a mediation model suggested that the rs2028122 genotype partially mediated the causal pathway of sleep duration leading to the development of PD on a positive effect.

Despite a recent increase in studies concerning on whether long and/or short sleep duration are able to increase the risk of PD, there are still no consistent conclusions, and the results are required further replication in a larger sample size. Lysen et al. (2019) found that habitual short sleepers were significantly more likely to develop PD when compared to those habitual suitable sleepers; this relationship was not observed in habitual long sleepers. These findings were further confirmed in an independent external cohort (Kay et al., 2018). However, Chen et al. (2006) found that a habitual long sleep duration may be an earlier biomarker of PD among female nurses. In another study, Postuma et al. (2017) reported that a self-reported long sleep duration increased the risk for non-PD conversion to PD. In this study, we discovered that both long and short sleep duration increased the risk of PD, even after adjusting for age, sex, socioeconomic status, sleep status, lifestyle, and physical activity. Our sensitivity analyses also exhibited a consistent finding among those participants who were worn the actigraphy devices. The sample size of our prospective study was the largest among these studies, which may be one of the main reasons why both long and short sleep duration were identified as the risk factors. Furthermore, our analyses showed that the association between sleep duration and PD risk appears to vary across RORA rs2028122 genotypes, and that the rs2028122 genotype partially mediates the causal pathway of sleep duration leading to the development of PD on a positive effect. These findings suggest that the previously inconsistent findings about the association of long and short sleep duration with the risk of PD may be influenced by the specific type of gene mutation level.

The most striking and interesting finding was that the association between sleep duration and PD risk varied across different RORA rs2028122 genotypes. Circadian rhythm disorder has been associated with changes in habitual sleep duration (Rutenfranz et al., 1988) and may increase the risk of PD (Leng et al., 2019). The RORA gene is involved in the regulation of circadian rhythms (Jetten, 2009) and has been considered as a novel locus for sleep duration (Hou et al., 2020). In our study, the rs2028122 genotype partially mediated the causal pathway of sleep duration leading to the development of PD on a positive effect. These findings highlight the role of the RORA gene in the regulation of sleep duration and PD; however, such finding has not been studied. Previous studies mainly focused on the association between the RORA gene and mental disorders, and the physiological function and physiopathological role of the rs2028122 genotypes remained unknown. Our study is the first to comprehensively investigate whether and how the RORA rs2028122 genotype and long/short sleep duration affect the risk of PD. The RORA SNP rs2028122 allele A has been shown to have a dominant protective effect against sleep disorders in patients with depression (Lavebratt et al., 2010). In this study, we found that the RORA rs2028122 mutation did not increase the risk of PD among normal sleepers, and neither long nor short sleep duration increased the risk of PD among carriers of AA genotype. However, the interaction between the rs2028122 mutations and long/short sleep duration appears to lead to a variable risk of PD, especially for habitual long sleepers in carriers of AG genotype and habitual short sleepers in carriers of GG genotype. The exact reasons for such interactions need to be investigated further.

There are several limitations to our study that should be addressed. First, participants in the UK Biobank have a restricted age range; therefore, our data cannot represent the entire population (Wang et al., 2021). Second, our study was limited by its cross-sectional design. Thus, causality cannot be inferred from the associations observed. Third, the physiological function and physiopathological role of the rs2028122 genotype need to be investigated further.

## Conclusion

In conclusion, our study demonstrated that long and short sleep duration, respectively, increased the risk of PD. The association between circadian profile and PD risk varied across different RORA rs2028122 genotypes. The RORA G risk allele, long sleep duration, and short sleep duration were identified as the risk factors of PD. Carriers of RORA rs2028122 AG and GG genotypes should alter their circadian profiles to reduce their risk of developing PD. A habitual short sleep duration is not recommended for carriers of rs2028122 GG genotype. Neither short nor long sleep duration are recommended for carriers of rs2028122 AG genotype. Our findings could help individuals to identify their potential risk profiles of certain individuals and provide guidance for individuals to take precise and timely actions to prevent the future incidence of PD. Public health guidance should focus on reducing the risk of PD by advocating healthy sleep habits.

## Data Availability Statement

The datasets presented in this study can be found in online repositories. The names of the repository/repositories and accession number(s) can be found on UK Biobank: https://biobank.ndph.ox.ac.uk/ukb/.

## Ethics Statement

The studies involving human participants were reviewed and approved by the Northwest Multi-Centre Research Ethics Committee (REC reference: 16/NW/0274). The patients/participants provided their written informed consent to participate in this study.

## Author Contributions

X-JD and YW had the idea for and designed this study, had full access to all the data in this study, take responsibility for the integrity of the data and the accuracy of the data analysis, and critically revised the manuscript for important intellectual content and gave final approval for the version to be published. YS and X-JD drafted the manuscript and did the analysis. JW took responsibility for double check of the data analysis. All authors agreed to be accountable for all the aspects of the work in ensuring that questions related to the accuracy or integrity of any part of the work are appropriately resolved.

## Conflict of Interest

The authors declare that the research was conducted in the absence of any commercial or financial relationships that could be construed as a potential conflict of interest.

## Publisher’s Note

All claims expressed in this article are solely those of the authors and do not necessarily represent those of their affiliated organizations, or those of the publisher, the editors and the reviewers. Any product that may be evaluated in this article, or claim that may be made by its manufacturer, is not guaranteed or endorsed by the publisher.
